# Long-term efficacy and safety of XEN-45 gel stent implantation in patients with normal-tension glaucoma

**DOI:** 10.1186/s12886-024-03522-6

**Published:** 2024-06-21

**Authors:** Emil Nasyrov, Caroline J. Gassel, David A. Merle, Jonas Neubauer, Bogomil Voykov

**Affiliations:** Centre for Ophthalmology, University Hospital Tuebingen, Elfriede-Aulhorn-Str. 7, 72076 Tuebingen, Germany

**Keywords:** Normal-tension glaucoma, Filtering surgery, Minimally invasive glaucoma surgery, MIGS, MIBS, Gel stent, Needling

## Abstract

**Background:**

Minimally invasive bleb surgery using the XEN-45 gel stent has not been established for the treatment of normal-tension glaucoma (NTG). The main objective of this study was to evaluate the long-term treatment efficacy and safety of XEN-45 in eyes with uncontrolled NTG.

**Methods:**

A retrospective analysis of patients with NTG who underwent XEN-45 gel stent implantation at university hospital Tuebingen between 2016 and 2021. The primary outcome measure was surgical success after three years defined as lowering of intraocular pressure (IOP) of ≥ 20%, with target IOP between 6 and 15 mmHg. Success was complete without and qualified irrespective of topical antiglaucoma medication use. The need for further glaucoma surgery, except for needling, was regarded as a failure. The secondary outcome measures included changes in mean IOP, number of antiglaucoma medications, and needling and complication rates.

**Results:**

Twenty-eight eyes from 23 patients were included in the final analysis. Complete and qualified success rates were 56.5% and 75% after three years, respectively. Mean postoperative IOP ± standard deviation decreased significantly after three years from 19.3 ± 2.0 mmHg at baseline to 13.7 ± 4.2 mmHg (*n* = 22; *p* < 0.0001). The median number of antiglaucoma medications decreased from 2 (range 0–4) to 0 after three years (range 0–3; *p* < 0.0001). Sixteen eyes (57%) required a median of 1 (range 1–3) needling procedures. One eye required further glaucoma surgery. No sight-threatening complications were observed.

**Conclusion:**

The XEN-45 stent is effective and safe for the long-term treatment of NTG. However, needling was frequently required to improve outcomes.

**Supplementary Information:**

The online version contains supplementary material available at 10.1186/s12886-024-03522-6.

## Introduction

Normal-tension glaucoma (NTG) is observed in approximately 30% of Caucasian patients with open-angle glaucoma (OAG) and with a higher prevalence in Asian populations, reported as high as 92% in Japanese OAG patients [1]. Lowering of intraocular pressure (IOP) is the only therapeutically modifiable factor in NTG patients [2, 3]. Trabeculectomy, regarded as the gold standard for glaucoma surgery, has demonstrated efficacy in reducing IOP levels in NTG patients. However, the procedure is associated with several potentially sight-threatening complications, such as hypotony maculopathy and bleb-associated endophthalmitis [1, 4–7]. Minimally invasive bleb surgery (MIBS) using the XEN-45 gel stent has been introduced in an attempt to reduce the risks associated with filtration surgery. An increasing number of studies have demonstrated that this stent is effective and safe for the treatment of various types of OAG [8–10]. However, most of these studies have investigated heterogeneous types of glaucoma and have only included small numbers of patients with NTG [8, 11, 12]. To the authors’ knowledge, only one published study has presented data on a sub-group of five NTG patients involving a 12-month follow-up [13]. Therefore, the role of the XEN-45 gel stent for the treatment of NTG patients has yet to be established. This study aimed to evaluate the long-term efficacy and safety of XEN-45 implantation in NTG patients.

## Methods

This was a retrospective, consecutive, non-comparative case series of patients with NTG who underwent XEN-45 gel stent implantation (Allergan, an AbbVie company, Illinois, USA) at a single tertiary centre, university hospital Tuebingen, between 2016 and 2021. NTG was defined as an OAG with IOP consistently less than 21 mmHg in accordance with the EGS Guidelines [14]. Surgery was performed in patients who were considered off target IOP despite maximum tolerated medical therapy. Patients who had a follow-up period of fewer than 12 months were excluded. A history of previous incisional glaucoma surgery was not an exclusion criterion. Ethical approval was granted by the ethics committee of the Medical Faculty of the University of Tuebingen which waived the requirement for patient consent for data to be used in this study due to the retrospective design (project-number: 378/2021BO2). The study was conducted in accordance with the tenets of the Declaration of Helsinki.

### Surgical technique

Surgery was performed by a single experienced surgeon (BV) under topical anaesthesia using oxybuprocaine eye drops, as described previously [10] (supplemental video). A small 25 µl bubble of 0.2 mg/ml mitomycin C (MMC) resulting in 5 µg of MMC (four patients received 10 µg and one patient received 20 µg) was injected into the subconjunctival space at 5 mm from the limbus at the 12 o’clock position. The bleb was then gently moved over the superonasal area. A 20 G sideport knife was used for the main incision, which was done 1 mm inferotemporally into the clear cornea, as well as for a smaller side-port incision made 3 clock-hours from the first primary incision. The anterior chamber was filled with 0.55 ml of 1.0% sodium hyaluronate viscoelastic (HEALON®, Abbott Laboratories Inc. Abbott Park, Illinois, USA). The XEN-45-injector was inserted through the main incision into the anterior chamber, with the needle angled toward the superonasal quadrant under gonioscopic control. The needle then carefully penetrated the sclera into the subconjunctival space, 3.0 mm from the limbus. The stent was released, and the injector was withdrawn. Once the proper position of the stent was verified, the viscoelastic was washed out with a balanced salt solution, resulting in the formation of a filtering bleb. Finally, 0.1 ml of 1 mg cefuroxime (Cefuroxim-Saar, Chephasaar Chem.-pharm. Fabrik GmbH, Germany) was injected into the anterior chamber.

### Pre- and postoperative management

Baseline IOP was assessed under antiglaucoma medication, which was stopped two weeks prior to surgery. Unpreserved dexamethasone eye drops were administered four times daily one week before surgery. On the first postoperative day, administration of moxifloxacin eye drops began. These were administered four times daily for two weeks. Additionally, unpreserved 1.3 mg/ml dexamethasone eye drops (Dexa EDO®, Dr. Gerhard Mannchem.-pharm. Fabrik GmbH, Germany) were administered five times daily starting from postoperative day one and were tapered over 6–8 weeks.

If post-surgery IOP levels were considered inadequate and clinical signs of bleb scarring were detected, a needling procedure was attempted first instead of reinitiating antiglaucoma medication (supplemental video). Needling was performed in the operating theatre under topical anaesthesia using oxybuprocaine eye drops. Mepivacaine was injected with a 30 G needle next to the XEN gel stent with the needle penetrating the conjunctiva approximately 5 mm from the stent. The fibrotic tissue around the gel stent was disrupted via sweeping movements of the needle tip. The stent was gently moved sideward to ensure tip mobility. Following this, 25 µl of 0.2 mg/ml MMC (5 µg) was injected into the filtering bleb. After surgery, moxifloxacin eye drops were administered four times daily for three days. Additionally, unpreserved dexamethasone eye drops were administered, with the dose gradually reduced over five weeks.

### Study measures

Follow-up were carried out on the first two postoperative days, at postoperative months 1, 3 and 6, and years 1, 2, 3, 4 and 5. These involved a full ophthalmological examination, which included an assessment of best corrected visual acuity (BCVA), IOP measurement using Goldmann applanation tonometry, and slit lamp and fundus examination. Perimetry was performed using the Octopus 900 perimeter (Haag-Streit, Koeniz, Switzerland).

The primary outcome measure was surgical success after three years. Success was defined as a ≥ 20% reduction in IOP from baseline that was within the range of 6–15 mmHg, in accordance with the guidelines set by of the World Glaucoma Association [15]. Additional upper IOP targets were set as IOP ≤ 18 mmHg and ≤ 12 mmHg. If patients did not meet the success criteria at two consecutive visits starting from month one, failure was considered. This was recorded as a failure on the first visit in which the criteria were not met. Furthermore, for success to be considered complete, it had to be achieved without antiglaucoma medication; however, it was considered qualified, regardless of whether topical IOP lowering medication was used. Loss of light perception and/or the need for further glaucoma surgery (except for needling procedures [15]) were considered failures.

A stricter success definition of IOP reduction (≥ 30%) was applied for comparison with literature with differing criteria [4, 6, 7]. As five patients underwent bilateral surgery, an additional one-eye analysis was performed by only including the eye operated on first to account for bias. The secondary outcome measures included reduced mean IOP compared to baseline, the number of individual antiglaucoma agents used, BCVA, and the number of complications and needling interventions.

### Statistical analysis

Statistical analyses and data plotting were performed using Prism 8 (GraphPad Software, La Jolla, CA, USA). Surgical success was evaluated using the Kaplan-Meier survival estimates and differences between estimates using the Log-rank test. Postoperative changes in IOP were evaluated using the paired t-test, as the D’Agostino & Pearson omnibus normality test indicated a normal distribution of data. Changes in the number of antiglaucoma medications used and BCVA were analysed using the Wilcoxon matched-pairs signed rank test, as the D’Agostino & Pearson omnibus normality test did not indicate a normal distribution of data. A probability value of *p* < 0.05 was considered statistically significant.

## Results

### Study patients

The mean follow-up time was 45 ± 16 months (range: 13–70 months). Five eyes from five patients received combined phacoemulsification and XEN-45 gel stent implantation. One patient was excluded from further analysis due to a follow-up of less than 12 months. In total 28 eyes of 23 patients were included in the final analysis. The demographic and clinical characteristics of the patients are summarized in Table 1.

**Table 1:** Demographic and clinical characteristics and previous surgery history

Characteristic	*n* = 28 eyes
Age (years)	Mean ± SD = 66.8 ± 7.3
Female sex (%)	*n* = 20 (71%)
White ethnicity	*n* = 28 (100%)
Preoperative medicated IOP (mmHg)	Mean ± SD = 19.3 ± 2.0
Number of preoperative medications	Median 2 (range 0–4) Mean ± SD = 2.3 ± 1.3
Lens status	
Phakic Pseudophakic	*n* = 20 (71%) *n* = 8 (29%)
Combined operation with phacoemulsification	*n* = 5 (18%)
Preoperative visual field (MD in dB)	Mean ± SD = 7.7 ± 5.8 Median 6.2 (range 0.5–17.7)
**Previous surgery**	
None ALT SLT CPC Trabeculotomy Trabeculectomy	*n* = 21 *n* = 1 *n* = 2 *n* = 2 *n* = 2 (4 and 6 years prior to XEN) *n* = 1 (11 years prior to XEN)

The number of medications used was calculated based on individual active agents. dB = decibel; MD = mean defect; SD = standard deviation; ATL = argon laser trabeculoplasty; SLT = selective laser trabeculoplasty; CPC = cyclophotocoagulation

### Effectiveness

**Fig. 1:**
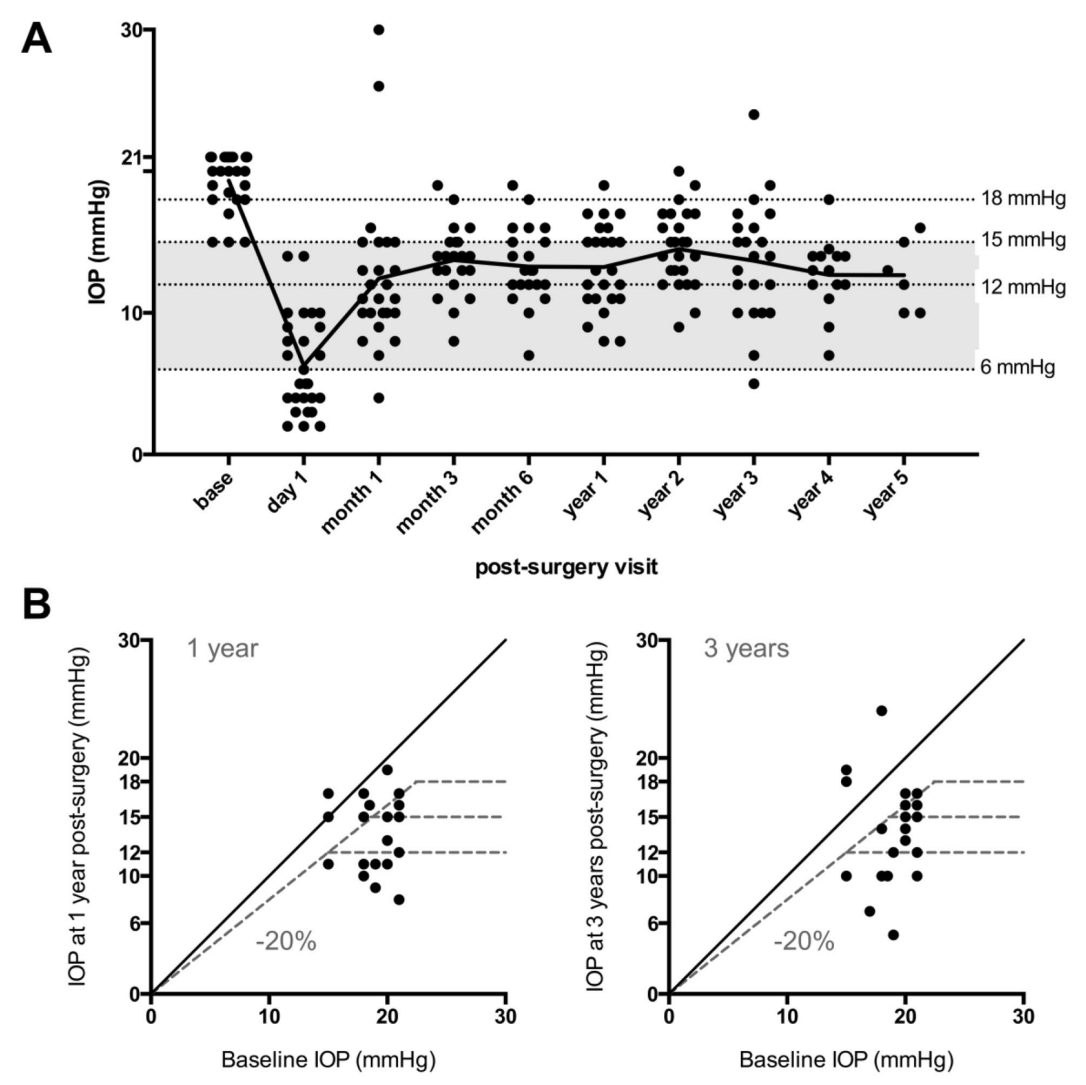
Evolution of postoperative mean intraocular pressure. Individual intraocular pressure (IOP) readings (**a**) are plotted across post-surgery visits. The graph delineates postoperative mean IOP development. Dotted horizontal lines demarcate the three IOP target zones (grey = primary outcome at 6 ≤ IOP ≤ 15 mmHg). IOP readings at one and three years (**b**) are compared to medicated baseline values. The diagonal line implies no change, the dotted diagonal line demarcates a 20% IOP reduction, and horizontals lines indicate post-surgery IOP targets

Preoperative mean IOP ± SD was significantly lowered from 19.3 ± 2.0 mmHg at baseline to 13.2 ± 3.0 mmHg one year and 14.5 ± 2.7 mmHg two years post-surgery, a 31.6% and 25% reduction, respectively (both *n* = 26; *p* < 0.0001, paired t-test) (Fig. 1A). Three years post-surgery, mean IOP was 13.7 ± 4.2. mmHg, a 29.3% reduction compared to baseline (*n* = 22; *p* < 0.0001) (Fig. 1A). Four years post-surgery, IOP levels had reduced by 34.4% compared to baseline (12.7 ± 2.6 mmHg; *n* = 14; *p* < 0.0001), while five years post-surgery a 34.5% reduction was observed (12.7 ± 2.5. mmHg; *n* = 6; *p* = 0.02) (Fig. 1A). No significant differences were found between the visits starting from one-month post-surgery (Fig. 1A). Figure 1B depicts the individual IOP readings from the one- and three-year follow-ups compared to baseline values.

**Fig. 2:**
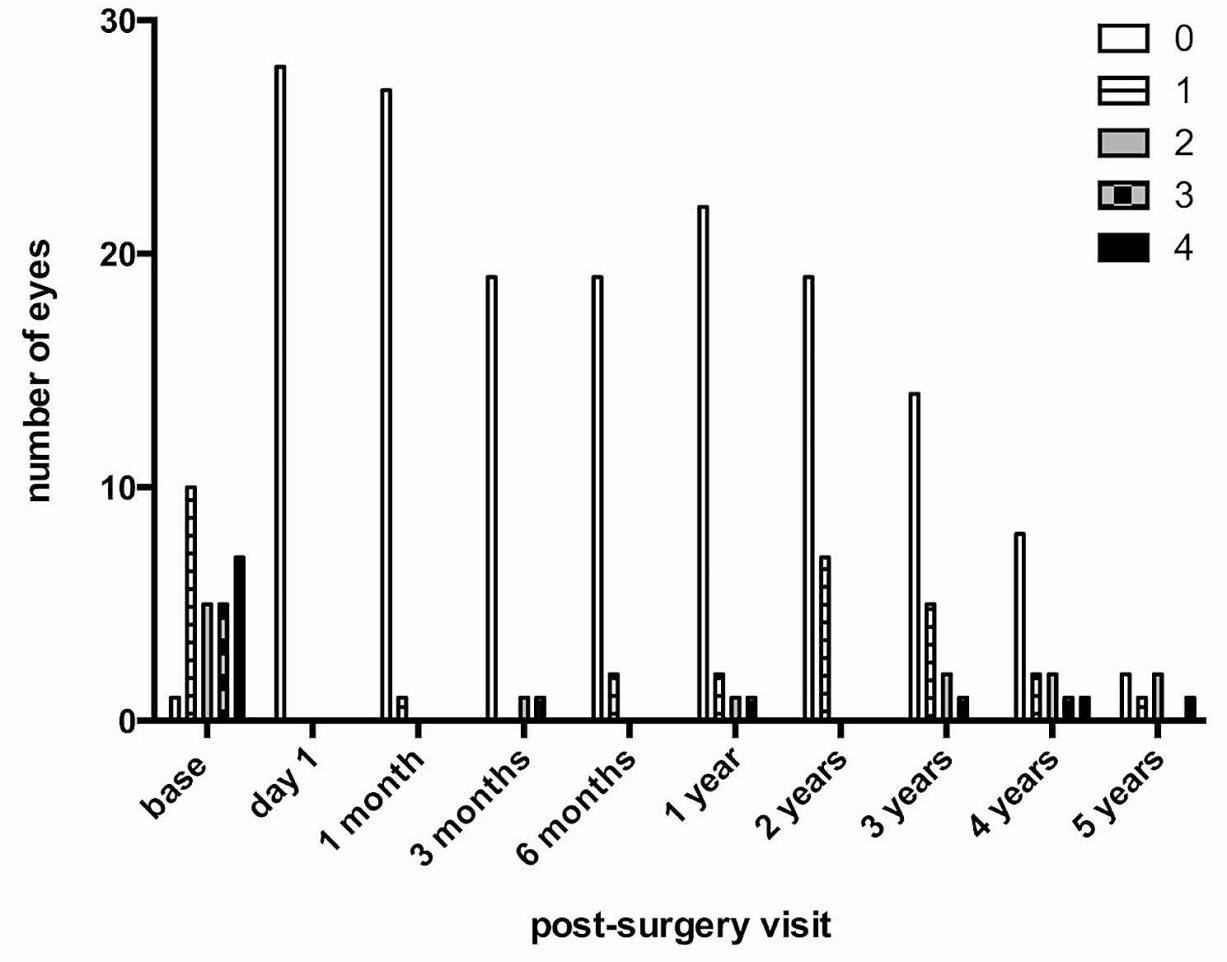
The number of patients using individual topical antiglaucoma medication. The legend indicates the number (0–4) of individual active antiglaucoma agents being used at post-surgery visits. No systemic intraocular pressure lowering medication was indicated throughout the post-surgery observation

The median number of antiglaucoma agents was significantly reduced from 2 at baseline (range 0–4, mean ± SD 2.3 ± 1.3) to 0 one year post-surgery (range 0–3; mean ± SD 0.3 ± 0.7; *p* < 0.0001, Wilcoxon matched-pairs signed rank test), 0 two years post-surgery (range 0–1; mean ± SD 0.3 ± 0.5; *p* < 0.0001), 0 three years post-surgery (range 0–3; mean ± SD 0.6 ± 0.9; *p* < 0.0001), and 0 four years post surgery (range 0–4; mean ± SD 0.9 ± 1.3; *p* = 0.031) (Fig. 2). However, a significant reduction was not seen five years post-surgery, with 1.5 agents (range 0–4; mean ± SD 1.5 ± 1.5; *p* = 0.4375) (Fig. 2). No patient required systemic carbonic anhydrase inhibitors during post-surgery observation.

**Fig. 3:**
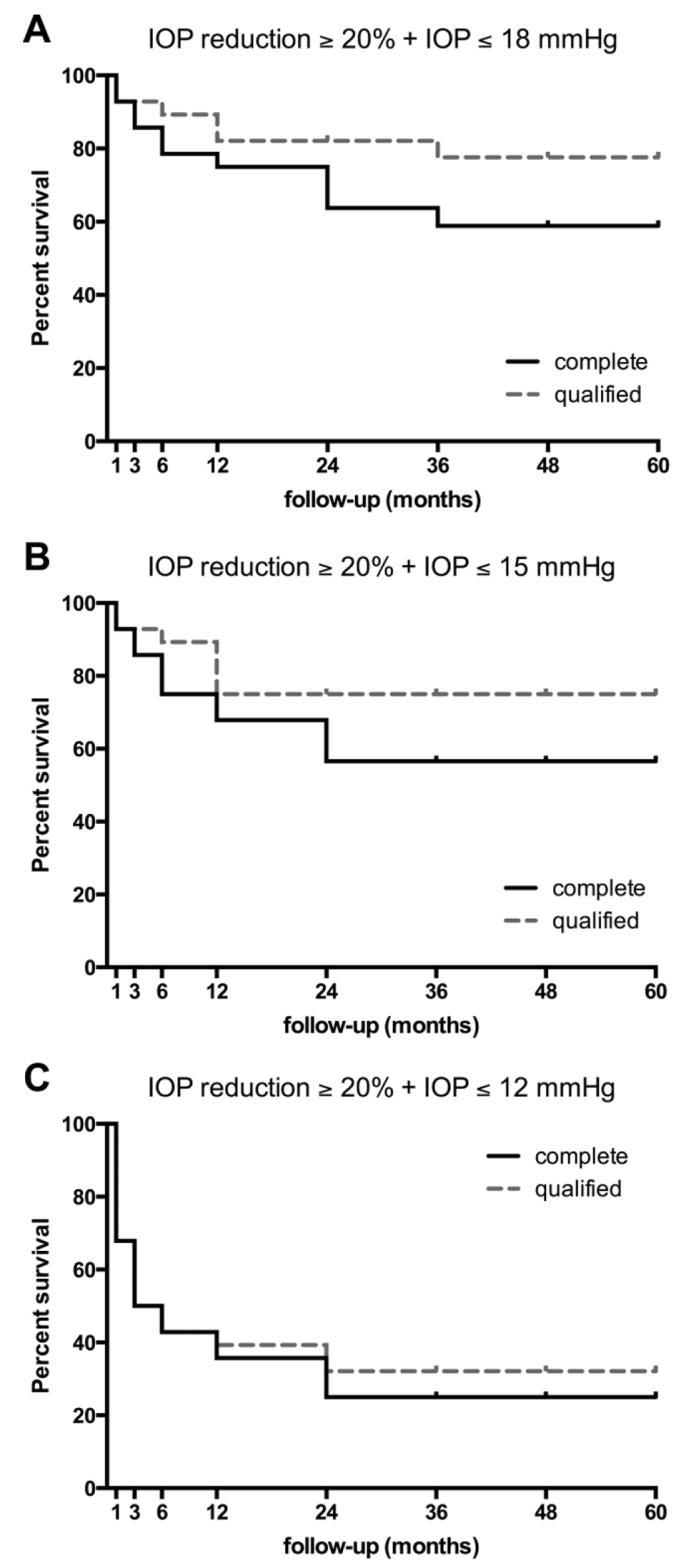
Kaplan-Meier survival probabilities for surgical success. Surgical success was defined as less than two consecutive intraocular pressure readings without a ≥ 20% reduction from baseline and target values within (**A**) ≥ 6 mmHg and ≤ 18 mmHg, (**B**) ≥ 6 mmHg and ≤ 15 mmHg and (**C**) ≥ 6 mmHg and ≤ 12 mmHg. Success was considered complete without and qualified irrespective of the use of additional antiglaucoma medication

The complete success rate with an IOP target of ≤ 15 mmHg was 67.9% at year one and 56.5% at years 2–5 post-surgery (Fig. 3B). The qualified success rate was 75% at years 1–5 post-surgery (Fig. 3B). The complete success rate with an IOP target of ≤ 18 mmHg was 75% at year one, 63.8% at year two, and 58.8% at years 3–5 (Fig. 3A). Qualified success rates were 82.1% at years 1–2 and 77.6% at years 3–5 (Fig. 3A). For the IOP target of ≤ 12 mmHg, complete and qualified success rates were 35.7% and 39.3% at year one, respectively (Fig. 3C). From years 2–5, these were 25% and 32.1%, respectively (Fig. 3C). To account for a possible introduction of bias by including the bilateral eyes of five patients, a one-eye analysis was performed for determining success rates (Fig. S1). The Log-rank test did not indicate any statistical differences regarding any success category between analyses. Neither IOP nor the number of medications used significantly differed (Tab. S1).

A stricter IOP reduction target of ≥ 30% and IOP target of ≤ 15 mmHg revealed complete success rates of 50% one year and 38.5% 2–4 years post-surgery (Fig. S2). Qualified success remained at 57.1% from years 1–5 (Fig. S2).

### Postoperative complications and interventions

Postoperative hyphema was observed in three eyes and resolved spontaneously (Table 2). Early hypotony with choroidal detachment was detected in one eye and resolved completely following a single injection of a cohesive viscoelastic into the anterior chamber. Long-term symptomatic hypotony was not observed. Twelve eyes (43%) did not require bleb intervention, while at least one needling procedure was performed on 16 eyes across 15 patients (57%; median 1, range 1–3) (Table 2). The median time to first needling was 125 days (range 12–1337), 386 days for the second (range 120–1895), and 931 days for the third (range 413–1122) (Table 2). Needling occurrence was highest in the first three months post-surgery (Table 2). Eyes that did not undergo or only underwent one needling procedure demonstrated similar success rates (Table 3). Eyes that underwent two procedures (*n* = 2) showed 50% success across all success categories, while eyes that underwent three procedures (*n* = 3) failed across all success categories during follow-up (Table 3). No patient required an incisional bleb revision. One eye required further surgical intervention via the implantation of a PreserFlo® MicroShunt (Table 2). This patient previously had three needlings and already failed across all success criteria within the first year post-surgery. Interestingly, patients with previously failed trabeculotomy (*n* = 2) or trabeculectomy (*n* = 1) did not require bleb interventions during follow-up. One patient with previous trabeculotomy failed after 6 months across all success categories, while the other two patients had complete success in regard to an IOP target of 6–15 mmHg after 3 years.

The mean BCVA ± SD was 0.12 ± 0.15 logMAR at baseline and did not significantly change after one year (0.13 ± 0.16; *p* = 0.8352, Wilcoxon matched-pairs signed rank test), two years (0.19 ± 0.3; *p* = 0.0603), four years (0.23 ± 0.53; *p* = 0.3633), or five years (0.13 ± 0.12; *p* = 0.25) post-surgery. However, a significant increase was seen three years post-surgery (0.2 ± 0.3; *p* = 0.0417). BCVA loss of ≥ 2 lines was recorded in 7 eyes (25%) attributable to cataract progression. No patient failed due to loss of light perception.

**Table 2:** Postoperative complications and interventions

Observed complication	Number of observed eyes (total *n* = 28)
Postoperative hyphema Early hypotony IOP spike (≥ 30 mmHg) Sustained hypotony Blebitis and endophthalmitis	*n* = 3 (11%) *n* = 1 (4%) *n* = 1 (4%) *n* = 0 (0%) *n* = 0 (0%)
**Needlings with MMC**	
Number of needlings per eye:	
0 ≥ 1 1 2 3 Overall	*n* = 12 (43%) *n* = 16 (57%) *n* = 11 (43%); median time to 1st event 125 d (range 12–1337) *n* = 2 (7%); median time to 2nd event 386 d (range 120–1895) *n* = 3 (11%); median to 3rd event 931 d (range 413–1122) *n* = 28; median 1 (range 1–3)
**Total number of needlings** < 3 months postop. 3–12 months postop 12–24 months postop. 24–36 months postop. 36–48 months postop. > 48 months postop.	*n* = 24 (100%) *n* = 9 (38%) *n* = 4 (17%) *n* = 4 (17%) *n* = 4 (17%) *n* = 2 (8%) *n* = 1 (4%)
**Further glaucoma surgery**	
PreserFlo MicroShunt implantation	*n* = 1 (4%); postoperative day 1673

Hypotony was defined as IOP < 6 mmHg. d = days; MMC = mitomycin C

**Table 3:** Association of needling events and success rates

	Success rates (%) based on an IOP lowering of ≥ 20% and upper IOP target of					
No. of needlings (no of eyes)	≤ 18 mmHg complete	≤ 18 mmHg qualified	≤ 15 mmHg complete	≤ 15 mmHg qualified	≤ 12 mmHg complete	≤ 12 mmHg qualified
0 (*n* = 12)	75.0	91.7	66.7	83.3	25.0	33.3
1 (*n* = 11)	63.6	90.9	63.6	90.9	27.3	36.4
2 (*n* = 2)	50.0	50.0	50.0	50.0	50.0	50.0
3 (*n* = 3)	0.0	0.0	0.0	0.0	0.0	0.0

Lower intraocular pressure (IOP) target range was IOP ≥ 6 mmHg across all categories. Complete success (without additional medication use); qualified success (regardless of additional medication use)

## Discussion

To the authors’ knowledge, this is the first study to evaluate the long-term efficacy and safety of the XEN-45 gel stent in a cohort of NTG patients. Three years after surgery, more than half of the studied eyes had achieved sustained IOP levels ≤ 15 mmHg without requiring IOP lowering medication. Three-quarters of the eyes achieved surgical success with further topical medication. Furthermore, one-fourth and one-third of the eyes achieved long-term complete and qualified success based on an IOP target of ≤ 12 mmHg. Most eyes (64%) did not require any topical IOP lowering medication three years post-surgery. Overall, our data indicates sustained IOP reduction and surgical success in NTG patients beyond three years after XEN-45 gel stent implantation.

Only one study by Schargus et al. had previously reported results on the XEN-45 gel stent, specifically in a sub-cohort of 5 NTG patients involving a 12-month follow-up [13]. Comparison of surgical success is difficult due to differences in study population and design, particularly regarding definitions of success criteria. Our cohort was slightly younger (66.3 ± 7.7 vs. 76.6 ± 3.9 years) and had higher mean IOP under maximally tolerated medication (19.3 ± 2.0 vs. 16.6 ± 3.4 mmHg). Furthermore, Schargus et al. defined success as IOP ≤ 15 mmHg and an IOP reduction of ≥ 40%, which differs form the criteria used in the current study. This might explain the lower complete and qualified success rates reported by Schargus et al., which were both 20% after one year, compared to our findings of 67.9% and 75%, respectively. However, the mean IOP reductions were comparable (31.6% in our study after one year vs. 29%) [13]. Additionally, both studies demonstrated a similar reduction in the use of topical medication after one year (Mean ± SD 2.3 ± 1.3 to 0.3 ± 0.7 vs. 2.6 ± 0.9 to 0.6 ± 0.9). Overall, our data supports the earlier findings by Schargus et al. and, for the first time, demonstrates sustained success beyond the first year post-XEN-45 implantation in NTG patients.

Regarding minimally invasive glaucoma surgery (MIGS), iStent was the most common approach used for NTG treatment among U.S. ophthalmologists between 2013 and 2018 [16]. Two retrospective studies reported a mean IOP reduction of 22% after one year and 13% after two years following combined phacoemulsification and iStent implantation [17, 18]. However, a study by Neuhann et al. only reported a significant IOP reduction for this combined procedure after one year, but not after two years [19]. Similarly, a prospective study on this combined operation in NTG patients found only a moderate IOP reduction of 9% after one year [20]. These result appear to be in accordance with another report on combined phacoemulsification and a variety of MIGS (not including XEN-45) resulting in an 11% IOP reduction, with only 5.4% of NTG patients achieving sustained surgical success (defined as an IOP reduction ≥ 30%) after 1.5 years [21]. In comparison, our study demonstrated that 50% and 38.5% of eyes achieved a ≥ 30% IOP reduction with an IOP target of ≤ 15 mmHg without further medication use 1 and 2–4 years after XEN-45 implantation, respectively.

Recent studies have demonstrated comparable success rates between the XEN-45 gel stent and trabeculectomy in OAG, however, trabeculectomy demonstrated numerically lower postoperative IOP levels [8, 9, 22]. Reported success rates of trabeculectomy in NTG patients (defined as an IOP reduction ≥ 30%) vary between 39.4% (qualified), 52% (complete and qualified) and 64.8% (complete) after three years [4, 6, 7]. To compare these results with our findings, we performed an additional analysis using stricter success criteria, namely an IOP reduction of ≥ 30% and target of ≤ 15 mmHg. Using this definition, our cohort achieved a complete and qualified success of 38.5% and 57.1% after three years, respectively. These are slightly lower compared to success rates reported in the aforementioned studies. However, the higher success rates of trabeculectomy are associated with a higher risk of postoperative complications. Several studies have demonstrated that higher IOP reduction via trabeculectomy might lead to a higher rate of over-filtration and late hypotony-associated complications in NTG patients, with late hypotony after trabeculectomy reported in 23.6%, 25%, and 30% of cases in the studies [5–7]. In comparison, we did not observe any long-term hypotony-related complications.

Rates of bleb-related complications after trabeculectomy such as endophthalmitis, blebitis, and late-onset bleb leak have been reported in up to 0–8%, 5–6%, and 3–6% of cases, respectively [4–7]. In comparison, we did not observe any bleb-related complications. In a recent review, Leung and Tham, suggested that minimally invasive procedures might present a safer option compared to traditional trabeculectomy for NTG specifically [1]. Our findings demonstrated that MIBS with the XEN-45 gel stent is a safe procedure in NTG patients.

Postoperative bleb modification is an important part of any filtering procedure. Needling rates of up to 14% have been reported following trabeculectomy in NTG patients [4, 6]. In our study, we performed at least one needling procedure in 57% of eyes, which was similar to or slightly higher than the rates reported for XEN-45 implantation in OAG [11–13, 23, 24]. However, comparison of needling rates is difficult since the indication to perform the procedure might differ greatly among surgeons and study populations. In our case, the threshold for indication was low, and needling was always preferred to using antiglaucoma medications if signs of bleb scarring were evident. Still, compared to trabeculectomy our needling rates were markedly higher, and patients should be advised preoperatively about the increased possibility of a needling procedure. Primary needlings at the time of XEN implantation could be a measure to reduce postoperative needling rates. However, outcomes of primary needling in OAG are controversial with Kerr et al. demonstrating a significant reduction of secondary needlings and Buenasmañanas-Maeso et al. finding no differences between eyes with or without primary needling [25, 26]. Additionally, the concentration of MMC used during XEN implantation could influence the needling rate. However, our unpublished data suggest no significant differences in needling rates between patients who received 5, 10, or 20 µg of MMC (manuscript submitted).

Interestingly, eyes that underwent only one needling procedure demonstrated comparable success rates to non-needled eyes. However, out data indicates that needing two or more needling procedures might be a negative predictor for sustained bleb function. While our evidence is weak due to the low number of patients, performing an incisional bleb revision may be a better option, if IOP levels are not sufficiently regulated after two needlings rather than attempting a third. Pre-emptive needlings or incisional bleb revision could be another potential strategy to reduce the number of re-needlings. Reports showed that elevated day 1 IOP might be a predictor of necessity for revision surgery [27]. In regard to bleb morphology no clear criteria exist that could predict bleb failure, and the strategy for timing and extent of bleb revisions is based on the surgeon’s discretion. In our experience incisional revision should be preferred when the XEN is not visible under the conjunctiva at the slit lamp, as this indicates a fibrotic bleb [28].

Studies with larger cohorts are needed to reliably guide clinical-decision making in this matter. In accordance with previous studies on OAG, we found that high rates of needling procedures were necessary for maintaining surgical success in NTG patients.

We acknowledge that our study has several limitations due to its retrospective design. First, our cohort was slightly heterogeneous regarding the surgical procedures and included 23 eyes that underwent a stand-alone XEN-45 gel stent implantation and five eyes that underwent this with combined phacoemulsification. However, previous studies have reported no differences in IOP reduction or success rates between the two procedures [24, 29]. The limited number of eyes that underwent the combined procedure in our study precluded us from performing a subgroup analysis. Second, most of the eyes were treated with 5 µg MMC; however, several eyes received 10 µg (*n* = 4) or 20 µg (*n* = 1). The low number of patients that received higher doses meant it was not possible to explore the effect of MMC doses in the current study. However, our data revealed no dose-dependent difference in the long-term success rates for OAG patients (manuscript submitted).

In conclusion, our study demonstrated that the XEN-45 gel stent is a safe and effective option for the long-term treatment of patients with NTG. Needling procedures were necessary for 57% of eyes to achieve IOP control. Complications were transient, self-limiting, and not sight-threatening.

### Electronic supplementary material

Below is the link to the electronic supplementary material.

Supplementary Material 1

Supplementary Material 2

Supplementary Material 3

## Data Availability

The datasets analysed during the current study are available from the corresponding author on reasonable request.
