# Supplementary material for: Long-term efficacy and safety of XEN-45 gel stent implantation in patients with normal-tension glaucoma
Source: BMC Ophthalmol. 2024 Jun 21;24:264. doi: 10.1186/s12886-024-03522-6 (PMC11191175; doi:10.1186/s12886-024-03522-6)
Supplement: Supplementary file 1 — Supplementary Material 1 [file 12886_2024_3522_MOESM1_ESM.docx]

**Supplementary Information**

**

**

**Supplemental Fig. 1** Comparison of survival probabilities between all-eyes and one-eye analyses

The second eyes of five patients who underwent bilateral XEN implantation were excluded from the one-eye analysis. Surgical success was defined as less than two consecutive intraocular pressure readings without a ≥ 20% reduction from baseline and target values within (**A** and **B**) ≥ 6 mmHg and ≤ 18 mmHg, (**C** and **D**) ≥ 6 mmHg and ≤ 15 mmHg and (**E** and **F**) ≥ 6 mmHg and ≤ 12 mmHg. Success was considered complete without and qualified irrespective of additional antiglaucoma medication use. The Log-rank test did not indicate any statistical differences between the analyses (A: p = 0.8156; B: p = 0.9976; C: p = 0.9939; D: p = 0.7772; E: p = 0.8362; and F: p= 0.7966).


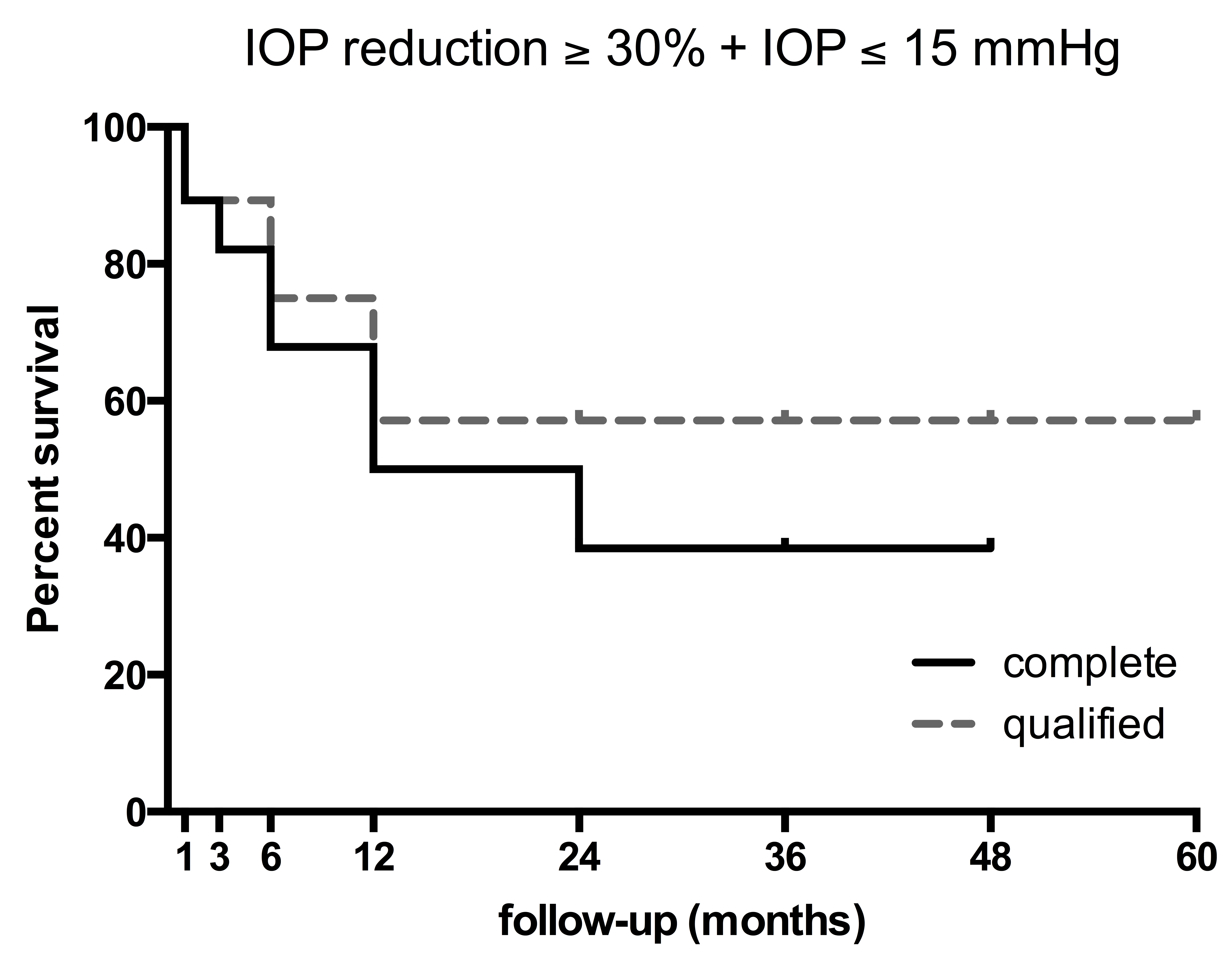


**Supplemental Fig. 2** Survival probabilities for success defined as an intraocular pressure reduction of ≥ 30%

The Kaplan-Meier survival probabilities for surgical success, defined as less than two consecutive intraocular pressure readings without a ≥ 30% reduction from baseline and target values within ≥ 6 mmHg and ≤ 15 mmHg. Success was considered complete without and qualified irrespective of additional antiglaucoma medication use. IOP = intraocular pressure.

**Supplemental Table 1** Comparison of postoperative mean intraocular pressure and medication use between all-eyes and one-eye analyses

| **IOP** | | | | | | | | | | |
| --- | --- | --- | --- | --- | --- | --- | --- | --- | --- | --- |
|  | **base** | **1 d** | **1 m** | **3 m** | **6 m** | **12 m** | **24 m** | **36 m** | **48 m** | **60 m** |
| n=28; mean IOP ± SD (mmHg) | 19.3  ±2.0 | 6.3  ±3.4 | 12.4  ±5.3 | 13.7  ±2.5 | 13.3  ± 2.7 | 13.2  ±3.0 | 14.5  ±2.7 | 13.7  ±4.2 | 12.7  ±2.6 | 12.7  ±2.5 |
| Mean IOP reduction to base (%) | / | 67.5 | 35.7 | 29.0 | 31.4 | 31.6 | 25.0 | 29.3 | 34.4 | 34.5 |
| n=23 (one-eye); mean IOP ± SD (mmHg) | 19.2  ±1.8 | 6.3  ±3.5 | 12.6  ±5.7 | 13.5  ±2.4 | 13.4  ±2.9 | 13.6  ±2.8 | 14.7  ±2.7 | 13.8  ±4.4 | 12.5  ±2.8 | 12.7  ±2.5 |
| Mean IOP reduction from baseline (%) | / | 67.5 | 34.7 | 30.0 | 30.2 | 29.5 | 23.8 | 28.4 | 34.8 | 34.2 |
| **Number of antiglaucoma medication** | | | | | | | | | | |
| n=28; mean ± SD | 2.3  ±1.3 | 0 | 0  ±0.2 | 0.2  ±0.8 | 0.1  ±0.3 | 0.3  ±0.7 | 0.3  ±0.5 | 0.5  ±0.9 | 0.9  ±1.3 | 1.5  ±1.5 |
| n=23 (one-eye);  mean ± SD | 2.4  ±1.2 | 0 | 0  ±0.2 | 0.3  ±0.9 | 0.1  ±0.3 | 0.3  ±0.8 | 0.3  ±0.5 | 0.7  ±0.9 | 1.1  ±1.4 | 1.5  ±1.5 |

For one-eye analysis, the second eyes of five patients who underwent bilateral XEN45 gel stent implantation were excluded. IOP = intraocular pressure; SD = standard deviation; d = day; m = month/s.
